# Supplementary material for: 3D-structured supports create complete data sets for electron crystallography
Source: Nat Commun. 2019 Jul 25;10:3316. doi: 10.1038/s41467-019-11326-2 (PMC6658500; doi:10.1038/s41467-019-11326-2)
Supplement: Supplementary file 1 — Supplementary Information [file 41467_2019_11326_MOESM1_ESM.pdf]

Supplementary Information

3D-structured supports create complete data sets for electron crystallography

Wennmacher *et al.*

— Supplementary Information —

3D-structured supports create complete data sets for electron crystallography

Julian T. C. Wennmacher, Christian Zaubitzer, Teng Li, Yeon Kyoung Bahk, Jing Wang, Jeroen A. van Bokhoven, and Tim Gruene

Supplementary Note 1: Sensitivity of Grids to Electron Radiation

No modification of the grids under irradiation was observed during our experiments. Supplementary Figure 4A and B shows a still recording of a ZSM-5 crystal on a grid covered with nylon fibres. The time difference is 30s, which corresponds about to 1/2 of a normal data set. Supplementary Figure 4C displays the difference between A and B. The figure shows only noise and no features, demonstrating that no mechanical change occurred under irradiation. Supplementary Figure 4D-F are the positive control that the difference image reveals samples movement. D and E shows the same crystal under rotation, *i.e.* a deliberate motion. The difference between D and E, Supplementary Figure 4F, shows the expected features of both D and E. Radiation damage does occur on nylon fibres under very strong irradiation. This is shown in Supplementary Figure 4G-J. H and I are still images with 2 min. irradiation with spot size 3. This is about 8-fold higher intensity and more than twice the exposure duration than what we used for our experiments. The difference Supplementary Figure 4J highlights movements of the nylon fibres. Note that the grid used for Supplementary Figure 4G-J is a carbon free support, which leaves the effect more pronounced of the isolating nylon fibres.

Supplementary Note 2: Data statistics for ZSM5 data sets

Supplementary Tables 1–4 present data statistics for all data sets. The correct setting for the unit cell parameters was confirmed by solving the structure from each individual data set with the direct methods, in space group *Pnma*. For some data sets, an increased number of trials was required, i.e. the complete command line for the program SHELXT reads *e.g.*

```
#> shelxt x11_11 -s"Pnma" -m1000
```

Resolution was cut at the 0.1 % significance level of $CC_{1/2}$ [1]. Note that crystallographic data statistics have been developed primiarily based on X-ray data, and absolute numbers may not be directly transferable when X-ray radiation is exchanged by electron radiation. Furthermore, statistics like I/σ_I and even $CC_{1/2}$ become less reliable when both completeness and multiplicity are as low as they often are with electron diffraction data. This applies to practically all structures

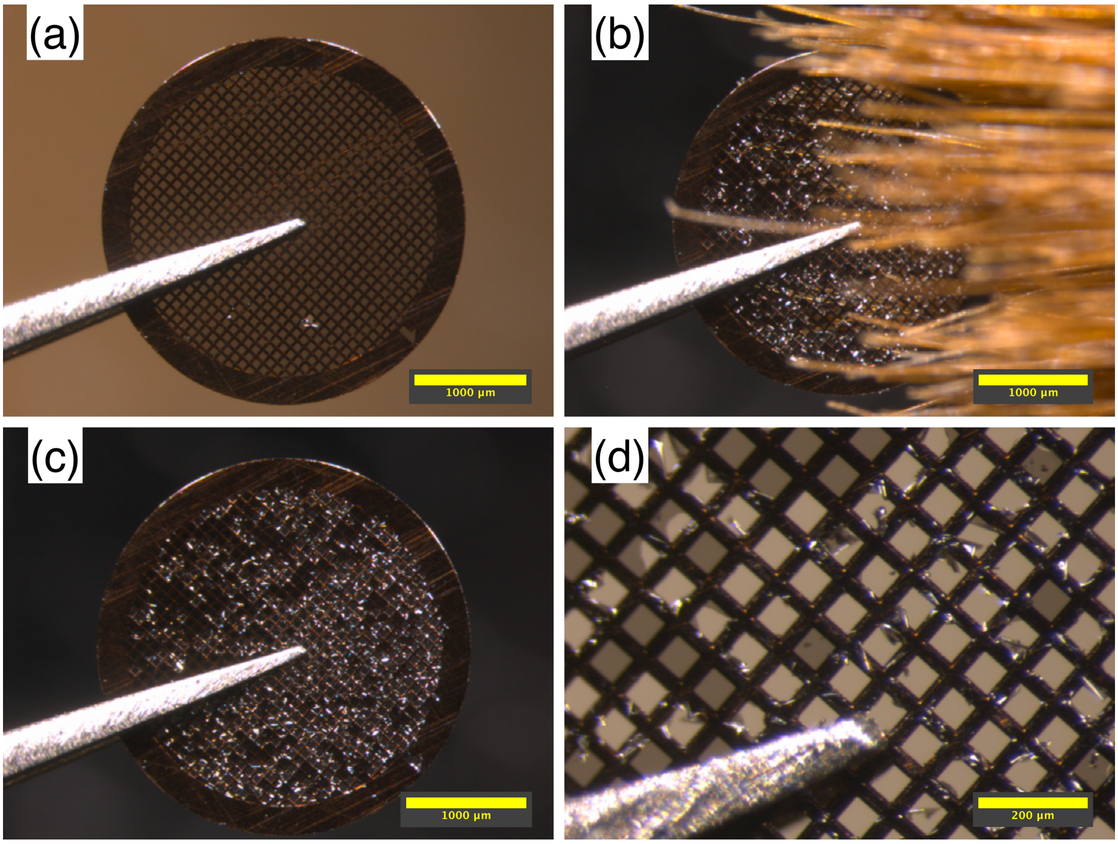

Supplementary Figure 1: Procedure for making coiled carbon film (a) A continuous carbon film TEM grid is clamped half between tweezers with the carbon film pointing upwards in diagonal orientation. (b) The grid is gently stroked in diagonal direction with a fine-haired paint brush. (c) Coiling creates nanometre and micrometre sized structures that interfere with light waves as observed from the brilliance of certain pixels. (d) Increasingly coiled carbon film can be achieved in random orientations with some meshes unaffected by the procedure.

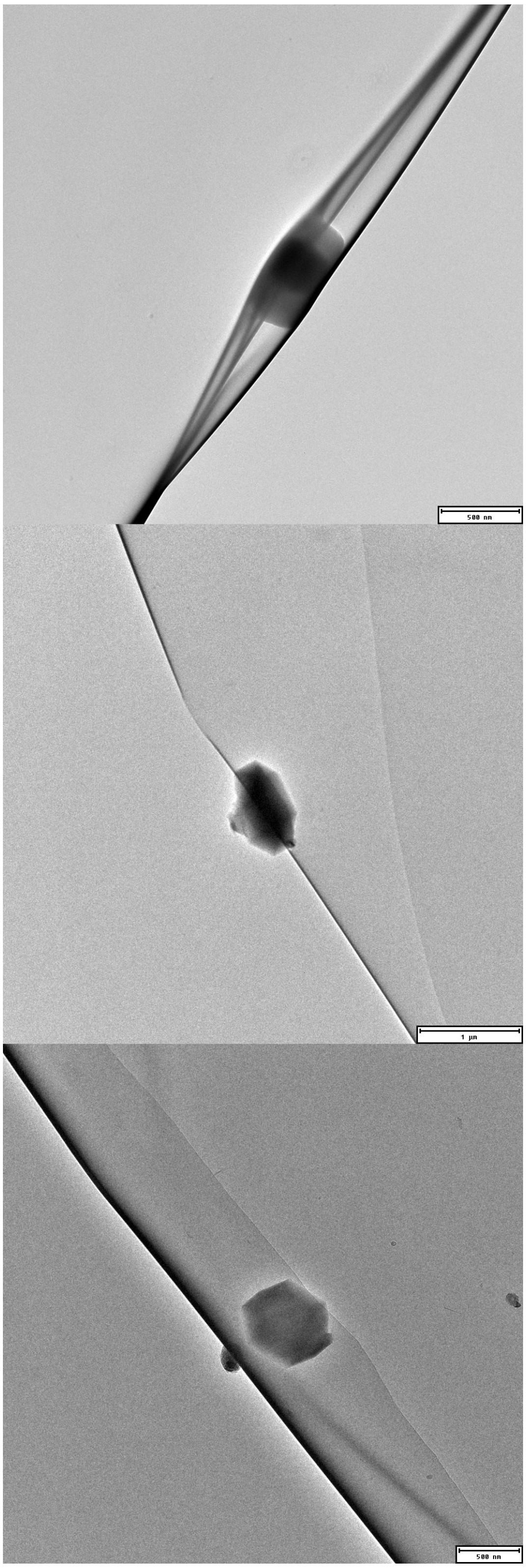

Supplementary Figure 2: Micrographs of ZSM-5 crystals in three different orientations sticking to the coiled carbon film.

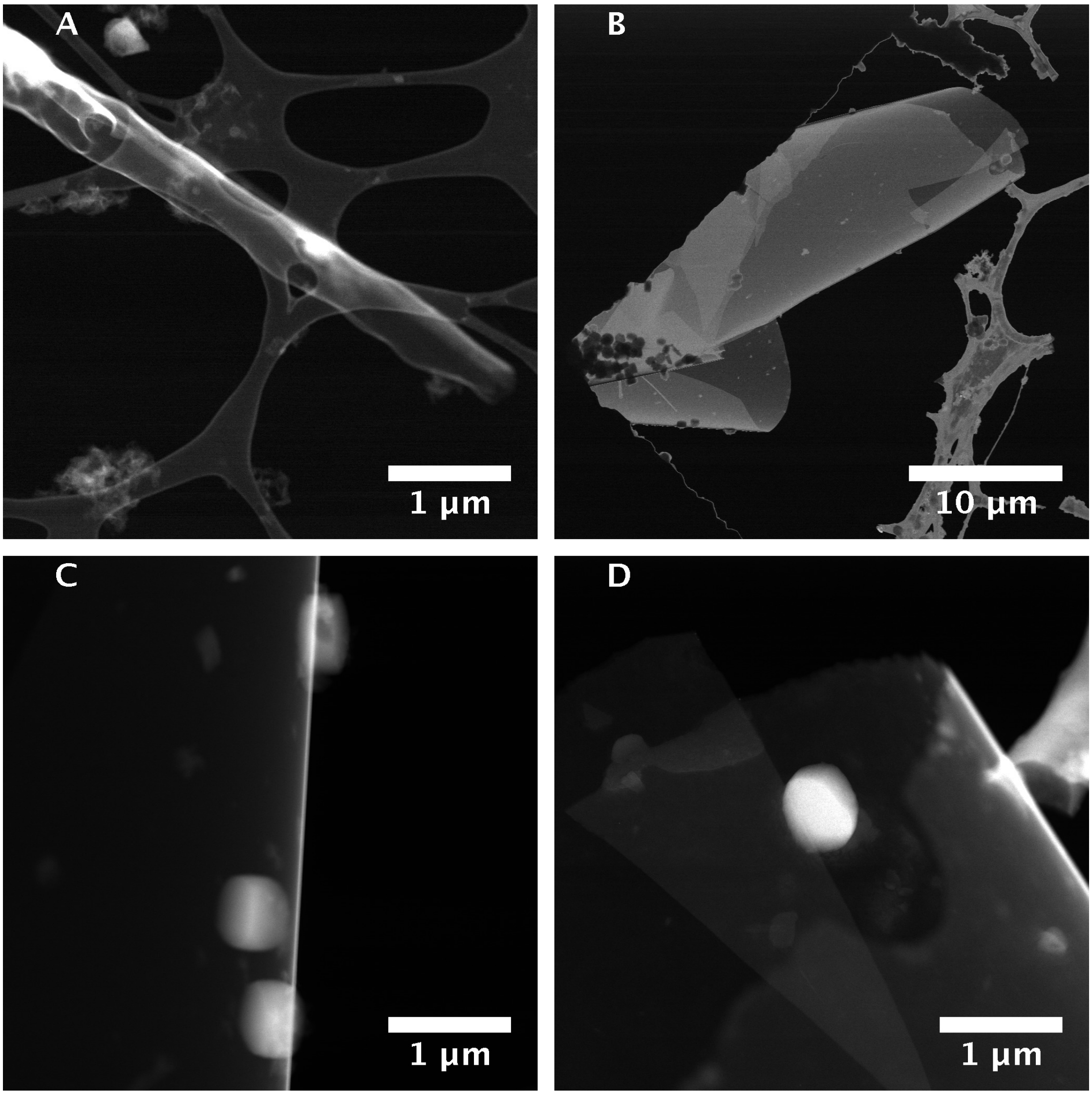

Supplementary Figure 3: The same folding protocol, that was used to produce the coiled carbon film was applied on a lacey carbon TEM grid covered with graphene. In *A* and *B* the graphene coils are shown. *C* and *D* The crystals follow the coils and change their orientation accordingly.

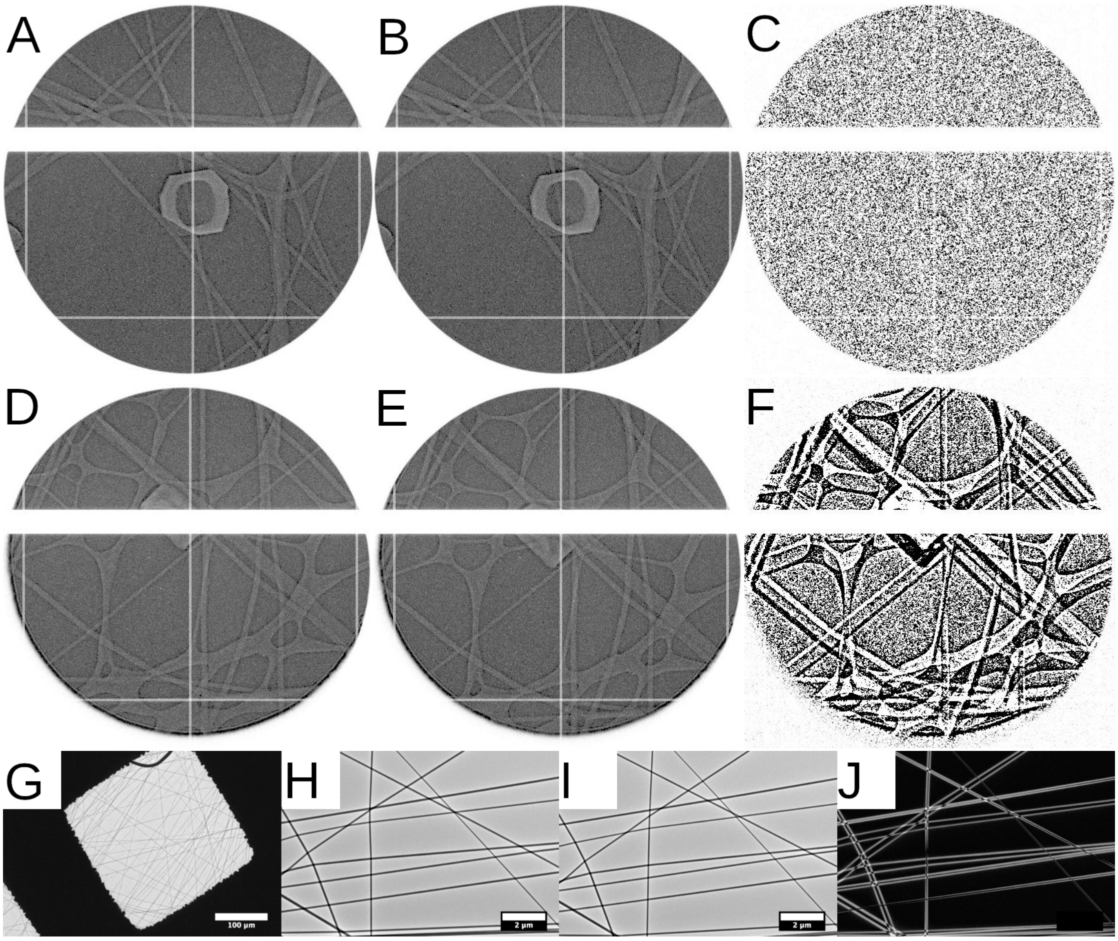

Supplementary Figure 4: Nylon fibres do not show alteration due to irradiation during data collection. A: image of zeolite crystal on lacey carbon grid covered with nylon fibres at time $t = 0$ s. B: same images as A at time $t = 30$ s. C: difference between A and B only shows noise. D-F: positive control, where the stage was deliberately moved. The difference image (F) shows features of both images. G-J: carbon-free grid covered with nylon fibres. G: large view of an entire mesh. H: 2000-fold magnification before irradiation. I: 2000-fold magnification after 2 min irradiation at spot size 3, *i.e.* 8-fold intensity compared with data collection conditions of images A-F. J: difference between J and I shows movement of the nylon fibres.

solved with electron diffraction, only some of which have been cited in this work [2, 3, 4, 5, 6, 7]. Dynamic refinement [2] is one way to improve the statistic of the R1-value, which measures the fit between model and observed data, but it will not improve the statistics that measure data quality. Furthermore, dynamic refinement is currently not available with integration software used for this study (priv. communication L. Palatinus). In general, the structure is the goal of the diffraction experiment and recent trends move away from classical quality indicators that primarily address precision rather than accuracy, and towards structure based quality assessment [8, 9]. In the case of the present work, both sets contain one data set of poor data quality. For the coiled carbon film, this is data set x10_5, for the nylon fibres, it is x11_11. Without these data sets, data completeness lacks about 1-2 reflections out of 800, and data completeness is only 99.9%. Once proper electron diffractometers have become more broadly accessible [10, 11, 12], such poor quality data sets can be replaced more easily with the collection of further data sets, as has been common practice in X-ray crystallography for many years.

References

[1] Karplus, P. A. & Diederichs, K. Linking crystallographic model and data quality. *Science* **336**, 1030–1033 (2012).

[2] Palatinus, L. *et al.* Hydrogen positions in single nanocrystals revealed by electron diffraction. *Science* **355**, 166–169 (2017).

[3] Simancas, J. *et al.* Ultrafast electron diffraction tomography for structure determination of the new zeolite ITQ-58. *J. Am. Chem. Soc.* **138**, 10116–10119 (2016).

[4] Wang, B. *et al.* A porous cobalt tetraphosphonate metal–organic framework: Accurate structure and guest molecule location determined by continuous-rotation electron diffraction. *Chem. - Eur. J.* **24**, 17429–17433 (2018).

[5] Duyvesteyn, H. M. E. *et al.* Machining protein microcrystals for structure determination by electron diffraction. *Proc. Natl. Acad. Sci. U. S. A.* **115**, 9569–9573 (2018).

[6] Das, P. P. *et al.* Crystal structures of two important pharmaceuticals solved by 3d precession electron diffraction tomography. *Org. Process Res. Dev.* **22**, 1365–1372 (2018).

[7] Gruene, T. *et al.* Rapid structure determination of microcrystalline molecular compounds using electron diffraction. *Angew. Chem., Int. Ed.* **57**, 16313–16317 (2018).

Supplementary Table 1: Statistical data of 6 MFI crystals sets acquired on the coiled carbon film. The data of the three crystals to reach data completeness are listed first. rr: resolution range. Data were cut above the 0.1% significance level of $CC_{1/2}$ in the highest resolution shell, except for x10_5 [1, 9], *cf.* Section Discussion and Supplementary Section B.

**Dataset ID**	x1_3	x2_4	x10_5	x3_5	x5_7	x6_8
$\Delta_{detector}$ [mm]	715	495	495	495	495	495
$\Delta\Phi$/frame [°]	0.0295					
total Φ [°]	130	130	130	125	80	90
space group	$P\bar{1}$					
unit cell axes [Å]						
a	19.941(8)	20.35(2)	20.33(2)	20.58(2)	20.36(3)	20.51(3)
b	19.812(5)	20.18(4)	20.23(1)	20.16(2)	20.10(2)	20.09(2)
c	13.229(2)	13.595(8)	13.72(2)	13.505(6)	13.629(4)	13.56(1)
α[°]	90.48(4)	90.88(8)	90.63(8)	90.72(8)	90.53(7)	89.18(5)
β[°]	90.43(6)	90.60(2)	90.66(6)	89.7(1)	89.63(6)	89.7(1)
γ[°]	90.1(1)	90.86(9)	91.54(5)	89.91(3)	89.15(8)	90.5(2)
# refl.	6,491	1,759	426	1,505	2,522	620
resolution [Å]						
full range	3.26–0.80	3.07–0.80	3.24–0.80	3.05–0.80	2.55–0.64	2.55–0.65
low shell	3.26–2.39	3.07–1.69	3.24–2.39	3.05–2.36	2.55–1.93	2.55–1.92
high shell	1.70–0.80	1.69–0.80	1.69–0.80	1.68–0.80	1.37–0.64	1.37–0.65
#refl. (mult.)						
full rr.	22,838 (1.5)	27,242 (1.7)	31,040 (1.8)	12,163 (1.7)	40,701 (1.8)	25,744 (1.7)
low rr.	1,128 (2)	987 (1.9)	1,269 (1.9)	461 (1.9)	1,402 (1.8)	922 (1.8)
high rr.	21,710 (1.5)	26,255 (1.7)	29,771(1.7)	11,702 (1.7)	39,299 (1.8)	24,822 (1.7)
#unique (compl.[%])						
full rr.	15,517 (73.1)	15,642 (68.5)	17,680 (76.5)	7,288 (31.5)	23,114 (53.0)	15,212 (35.2)
low rr.	577 (72.6)	517 (58.6)	660 (74.8)	244 (27.1)	782 (53.0)	515 (31.2)
high rr.	14,940 (73.2)	15,125 (68.7)	15174(76.5)	7,044 (31.5)	22,332 (53.1)	14,697 (35,2)
I/σ_I	2.2/8.0/2.4	1.9/8.9/2	0.6/2.7/0.6	1.3/4.9/1.4	1.6/7.4/1.8	1.0/4.8/1.1
$CC_{1/2}$[%]	99.0/99.1/68.4	99.1/99.4/76.0	85.2/94.7/23.7	97.5/97.8/76.0	99.1/99.1/69.0	97.3/97.1/64.3
R_{meas}[%]	12.8/7.6/80.6	27.0/7.2/73.2	132.6/26.6/469.7	40.2/16.1/89.4	23.0/7.8/103.0	38.6/12.7/201.4

Supplementary Table 2: Merged datasets acquired on the coiled carbon film. The first column contains the statistics from merging the first three data sets in Supplementary Table 1, the second column contains the statistics from merging for all six data sets.

**Dataset ID**	minimum (1–3)	all (1–6)
space group	$P\bar{1}$	
unit cell axes [Å]		
a	20.042(7)	20.111(6)
b	19.895(5)	19.9334(4)
c	13.288(2)	13.358(2)
α[°]	90.58(3)	90.30(2)
β[°]	90.55(4)	90.22(3)
γ[°]	91.07(3)	90.30(2)
resolution [Å]		
full range	20.0–1.05	20.1–1.05
low shell	20.0–4.70	20.1–4.70
high shell	1.08–1.05	1.08–1.05
#refl. (mult.)		
full res. range	37,696 (3.9)	56,138 (5.8)
low res. range	403 (4.1)	625 (6.0)
high res. range	2,879 (4.2)	4,287 (6.2)
#unique (compl.[%])		
full res. range	9,511 (99.3)	9,666 (99.9)
low res. range	99 (97.1)	105 (99.1)
high res. range	687 (100.0)	692 (100.0)
I/σ_I	1.6/2.3/1.2	1.6/1.8/1.2
$CC_{1/2}$[%]	83.8/70.7/21.8	83.1/93.1/35.0
R_{meas}[%]	66.0/58.9/179	75.2/70.2/156

Supplementary Table 3: Statistical data of the 6 ZSM-5 crystals sets acquired on the nylon three-dimensional network. The data of the four crystals to reach data completeness are located left to the double line. rr.: resolution range. Data were cut at the 0.1% significance level of $CC_{1/2}$ in the highest resolution shell [1, 9], except for x11_11, *cf.* Section Discussion and Supplementary Section B.

**Dataset ID**	x06_11	x08_5	x10_9	x11_11	x04_7	x09_7
$\Delta_{detector}$ [mm]	495					
$\Delta\Phi$/frame [°]	0.0293					
total Φ [°]	120	120	120	120	135	130
space group	$P\bar{1}$					
unit cell axes [Å]						
a	20.23(5)	20.57(2)	20.03(3)	20.42(2)	20.22(2)	20.50(1)
b	20.68(3)	19.818(5)	20.37(4)	20.20(1)	20.429(9)	20.35(2)
c	13.858(9)	13.67(1)	13.768(5)	13.76(1)	13.833(7)	13.435(9)
α[°]	90.1(1)	90.1(1)	90.11(3)	90.41(4)	90.67(2)	91.1(1)
β[°]	91.0(2)	90.40(5)	90.8(2)	90.34(6)	90.88(3)	91.19(8)
γ[°]	90.4(1)	90.4(1)	89.97(4)	91.77(6)	91.17(3)	90.34(5)
# refl.	1,619	4,570	15,749	3,317	12,467	5,007
resolution [Å]						
full range	20.2–1.00	14.3–0.70	13.8–0.65	20.4–1.00	20.4–0.65	20.5–0.90
low shell	20.2–2.96	14.3–2.08	13.8–1.93	20.4–2.96	20.4–1.94	20.5–2.7
high shell	1.06–1.00	0.74–0.70	0.69–0.65	1.06–1.00	1.38–0.65	0.95–0.90
#refl. (mult.)						
full rr.	13,688 (1.7)	35,545 (1.8)	47,439 (1.8)	13,110 (1.7)	44,406 (1.8)	15,454 (1.7)
low rr.	555 (1.9)	1,295 (1.9)	1779 (1.8)	570 (1.9)	1,615 (1.9)	624 (1.9)
high rr.	2,221 (1.8)	5,945 (1.8)	7,959 (1.8)	2,090 (1.7)	4,2791 (1.8)	2,571 (1.7)
#unique (compl.[%])						
full rr.	8,039 (65.4)	20,101 (58.4)	26,794 (62.6)	7,731 (64.4)	24,934 (57.4)	9,039 (55.7)
low rr.	292 (62.4)	691 (53.6)	971 (59.3)	298 (64.8)	868 (53.2)	329 (54.3)
high rr.	1,260 (63.8)	3,299 (59.5)	4,404 (63.5)	1,202 (61.5)	24,066 (57.2)	1,458 (55.9)
I/σ_I	1.7/5.3/0.7	3.7/10.9/1.5	2.4/6.0/1.1	0.9/2.2/0.4	2.1/5.2/2.3	3.1/7.7/1.7
$CC_{1/2}$[%]	95.7/96.3/48.1	99.1/99.4/85.4	98.0/98.7/80.1	84.5/92.2/17.9	97.5/97.7/91.2	98.9/98.6/86.5
R_{meas}[%]	31.5/14.7/87.6	11.1/6.5/46.5	17.7/10.3/54.4	47.3/23.6/139	20.5/13.5/33.5	15.5/9.7/46.1

Supplementary Table 4: Merged datasets acquired on the nylon fibres. The first column contains the statistics from merging the first four data sets in Supplementary Table 2, the second column contains the statistics from merging for all six data sets. Cell calculated as weighted mean from corresponding individual data sets

**Dataset ID**	data sets 1–4	data sets 1–7
space group	$P\bar{1}$	
unit cell axes [Å]		
a	20.29(1)	20.410(7)
b	19.955(7)	20.139(5)
c	13.786(4)	13.750(3)
α[°]	90.22(3)	90.47(2)
β[°]	90.45(5)	90.81(2)
γ[°]	90.59(3)	90.85(2)
resolution [Å]		
full range	20.3–1.05	20.4–1.05
low shell	20.3–4.7	20.4–4.70
high shell	1.08–1.05	1.08–1.05
#refl. (mult.)		
full res. range	41,356 (4.1)	60,598 (5.9)
low res. range	520 (4.8)	755 (6.9)
high res. range	3,255 (4.5)	4,907 (6.5)
#unique (compl.[%])		
full res. range	10,042 (99.5)	10,213 (99.9)
low res. range	109 (100.0)	110 (100.0)
high res. range	721 (99.7)	759 (100.0)
I/σ_I	2.5/3.2/2.2	2.8/3.5/2.3
$CC_{1/2}$[%]	82.4/81.5/76.8	84.2/77.1/67.8
R_{meas}[%]	54.1/44.9/73.9	54.3/39.2/72.2

[8] Chen, V. B. *et al.* *Molprobity*: all-atom structure validation for macromolecular crystallography. *Acta Crystallogr.* **66**, 12–21 (2010).

[9] Rupp, B. Against method: Table 1-*Cui Bono*? *Structure* **26**, 919–923 (2018).

[10] Cichocka, M. O., Ångström, J., Wang, B., Zou, X. & Smeets, S. High-throughput continuous rotation electron diffraction data acquisition *via* software automation. *J. Appl. Crystallogr.* **51**, 1652–1661 (2018).

[11] Yonekura, K., Ishikawa, T. & Maki-Yonekura, S. A new cryo-em system for electron 3d crystallography by eefd. *J. Struct. Biol.* **206**, 243–253 (2019).

[12] Heidler, J. *et al.* Design guidelines for an electron diffractometer for structural chemistry and structural biology. *Acta Crystallogr.* **D75**, 458–466 (2019).

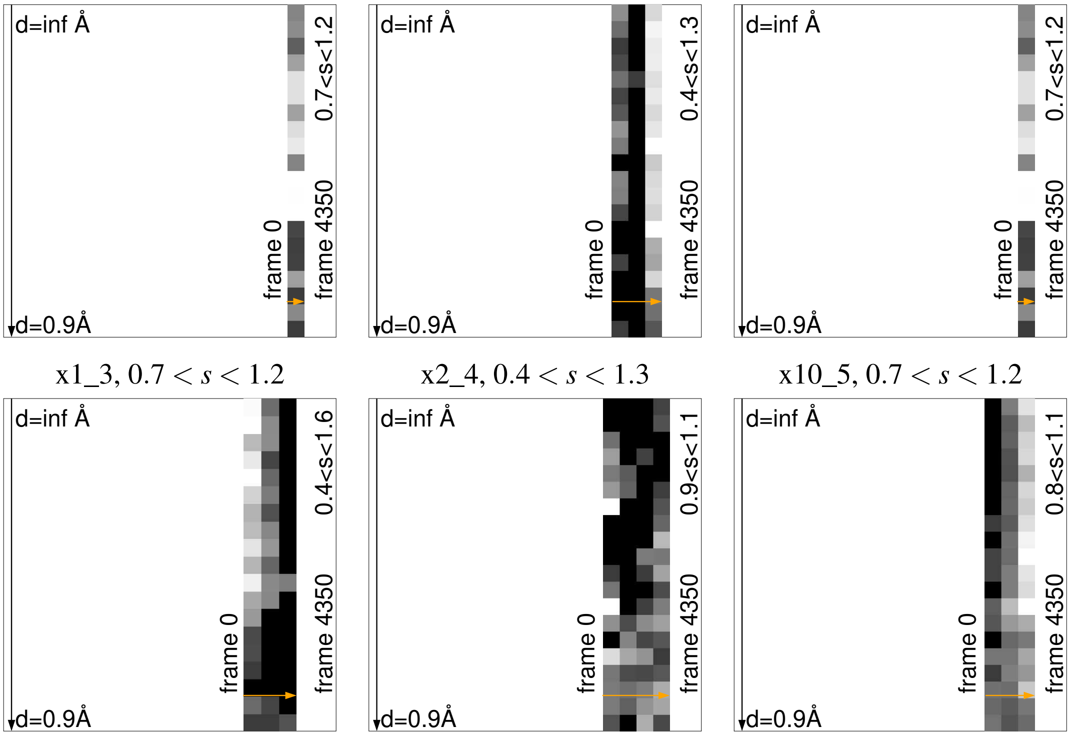

x1_3, $0.7 < s < 1.2$ x2_4, $0.4 < s < 1.3$ x10_5, $0.7 < s < 1.2$

x3_5, $0.4 < s < 1.6$ x5_7, $0.9 < s < 1.1$ x6_8, $0.8 < s < 1.1$

Supplementary Figure 5: Per-frame scaling factors s for the individual data sets from coiled grids, as listed in Table 1. Scale factor range from white (low scale factor s) to black (high scale factor s) The binning both in resolution (top to bottom) and in frame number (left to right) is set by XSCALE. The contrast of all figures is on the same scale, $0.5 =$ white, $1.5 =$ black.

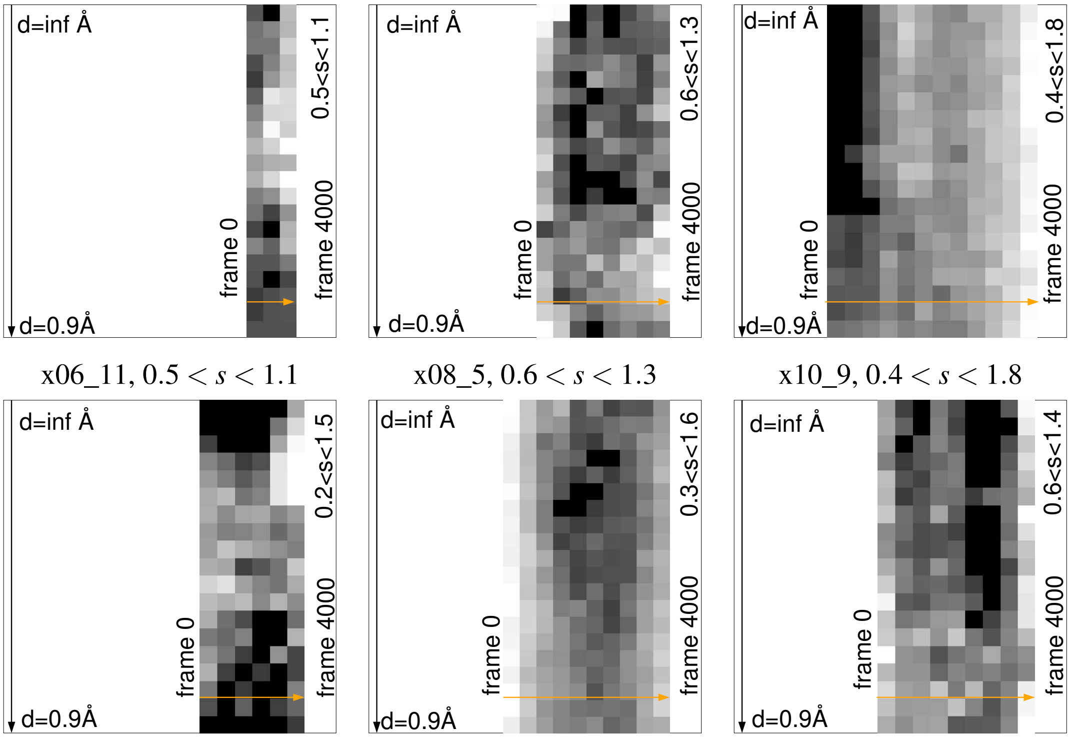

x06_11, $0.5 < s < 1.1$ | x08_5, $0.6 < s < 1.3$ | x10_9, $0.4 < s < 1.8$

x11_11, $0.2 < s < 1.5$ | x04_7, $0.3 < s < 1.6$ | x09_7, $0.6 < s < 1.4$

Supplementary Figure 6: Per-frame scaling factors s for the individual data sets from nylon covered grids, as listed in Table 3. Scale factor range from white (low scale factor s) to black (high scale factor s) The binning both in resolution (top to bottom) and in frame number (left to right) is set by XSCALE.
